# Supplementary material for: Extracellular Alterations in pH and K+ Modify the Murine Brain Endothelial Cell Total and Phospho-Proteome
Source: Pharmaceutics. 2022 Jul 15;14(7):1469. doi: 10.3390/pharmaceutics14071469 (PMC9324801; doi:10.3390/pharmaceutics14071469)
Supplement: Supplementary file 1 [file pharmaceutics-14-01469-s001.zip › Figure S1.pptx]

## Slide 1
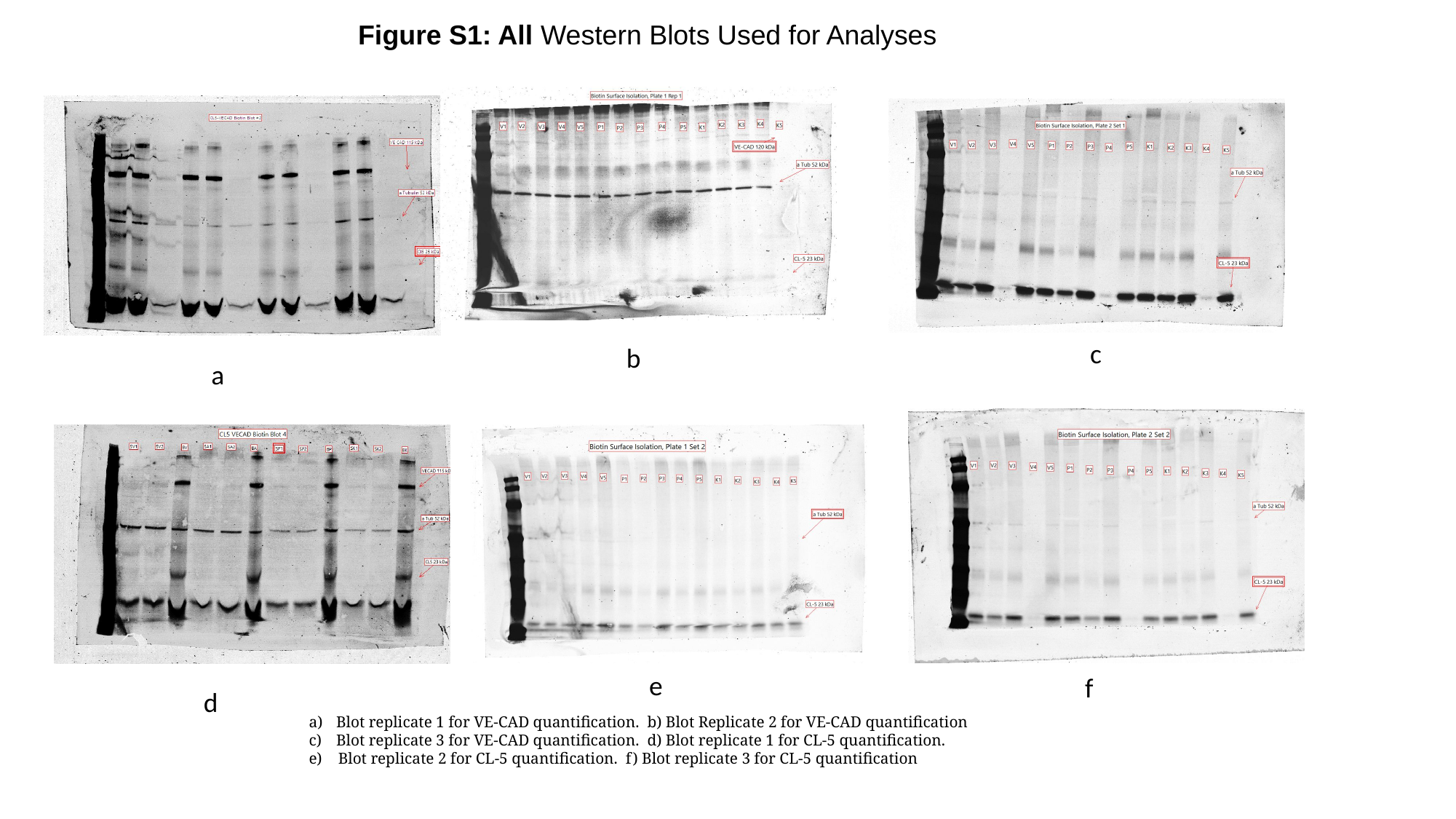

Figure S1: All Western Blots Used for Analyses
c
b
a
e
f
d
Blot replicate 1 for VE-CAD quantification. b) Blot Replicate 2 for VE-CAD quantification
Blot replicate 3 for VE-CAD quantification. d) Blot replicate 1 for CL-5 quantification.
e) Blot replicate 2 for CL-5 quantification. f) Blot replicate 3 for CL-5 quantification
